# Assessing Acute Pericarditis with T1 Mapping: A Supportive Contrast-Free CMR Marker

**DOI:** 10.3390/tomography10120137

**Published:** 2024-11-27

**Authors:** Riccardo Cau, Francesco Pisu, Roberta Montisci, Tommaso D’Angelo, Cesare Mantini, Rodrigo Salgado, Luca Saba

**Affiliations:** 1 Department of Radiology, Azienda Ospedaliero Universitaria (A.O.U.), di Cagliari—Polo di Monserrato s.s. 554 Monserrato, 09045 Cagliari, Italy; fra.pisu1@gmail.com; 2 Department of Cardiology, Azienda Ospedaliero Universitaria (A.O.U.), di Cagliari—Polo di Monserrato s.s. 554 Monserrato, 09045 Cagliari, Italy; rmontisci@unica.it; 3 Department of Biomedical Sciences and Morphological and Functional Imaging, G. Martino University Hospital, University of Messina, 98124 Messina, Italy; tommasodang@gmail.com; 4 Department of Radiology and Nuclear Medicine, Erasmus MC, Doctor Molewaterplein 40, 3015 GD Rotterdam, The Netherlands; 5 Department of Neuroscience, Imaging and Clinical Sciences, ‘G. d’Annunzio’ University, 66100 Chieti, Italy; cesare.mantini@gmail.com; 6 Department of Radiology, Universitair Ziekenhuis Antwerpen, 2650 Edegem, Belgium; rodrigo.salgado@uza.be

**Keywords:** pericarditis, magnetic resonance imaging, non-contrast, T1 mapping

## Abstract

Objective: The purpose of this study was to explore the impact of pericardial T1 mapping as a potential supportive non-contrast cardiovascular magnetic resonance (CMR) parameter in the diagnosis of acute pericarditis. Additionally, we investigated the relationship between T1 mapping values in acute pericarditis patients and their demographic data, cardiovascular risk factors, clinical parameters, cardiac biomarkers, and cardiac function. Method: This retrospective study included CMR scans in 35 consecutive patients with acute pericarditis (26 males, 45.54 ± 23.38 years). Moreover, we included 17 sex- and age-matched healthy controls (12 males, mean age 47.78 ±19.38 years). CMR-derived pericardial T1 mapping values, which included all pericardial structures within the pericardial layers—encompassing both pericardial effusion and pericardial layer thickness—were analyzed and compared between acute pericarditis patients and controls. Results: Compared to the matched control group, acute pericarditis patients demonstrated significantly lower pericardial T1 mapping values (2137 ms ± 519 vs. 3268 ms ± 362, *p* = 0.001). In the multivariable analysis, the pericardial T1 mapping value was independently associated with the severity of pericardial late gadolinium enhancement (LGE) (β coefficient = −3.271, *p* = 0.003). The receiver operating characteristic curve analysis showed that the diagnostic performance of pericardial T1 mapping in discriminating acute pericarditis patients was excellent, with an area under the curve of 0.97 (95% CI = 0.94–0.98), using a threshold of 2862.5 ms. Conclusions: Pericardial T1 mapping values could serve as an additional non-contrast CMR parameter for identifying patients with acute pericarditis, demonstrating an independent association with the severity of pericardial LGE.

## 1. Introduction

Acute pericarditis is an inflammation of the pericardium that can arise from various causes, including infections, autoimmune disorders, metabolic diseases, radiation exposure, and medical interventions [1,2].

According to the current European Society of Cardiology (ESC) guidelines, diagnosing pericarditis primarily depends on clinical symptoms, electrocardiogram (ECG) findings, and echocardiographic features. Transthoracic echocardiography is the primary imaging modality for patients with suspected acute pericarditis, providing essential information on pericardial layer thickening, hyperechogenicity, the presence and volume of pericardial effusion, and any associated hemodynamic effects. Additionally, echocardiography can offer a qualitative assessment of pericardial fluid, such as identifying fibrin strands or hemopericardium, which may suggest different etiologies [2].

Cardiovascular magnetic resonance (CMR) is suggested to provide confirmatory findings of an inflamed pericardium, rule out concomitant myocardial involvement (i.e., ‘myopericarditis’), exclude myocardial ischemia, and identify complications, thereby tailoring the therapy [2].

CMR is an excellent non-invasive imaging technique for assessing the pericardium’s anatomical and morphological characteristics. It is particularly useful for detecting the presence of edema and scarring due to its spatial resolution and advanced tissue characterization capabilities [3,4,5].

The recently introduced T1 mapping technique, which measures the longitudinal relaxation time determined by the rate at which protons return to their equilibrium state after being excited by a radiofrequency pulse, offers a sensitive and quantitative assessment of myocardial tissue characterization. This technique provides comprehensive tissue characterization information without the need for contrast media administration [6]. With the rapid increase in CMR examinations, utilizing CMR parameters from abbreviated protocols could greatly benefit real-life clinical practice [7,8,9,10]. Moreover, cardiac symptoms like orthopnea can reduce patient tolerance for CMR procedures, and renal disease may hamper contrast media administration.

T1 mapping has successfully been used to discriminate between transudate and exudate pericardial effusion, based on the paramagnetic properties of proteins and cells leaked into the inflamed pericardial fluid [11].

Currently, little is known about the impact of T1 mapping in patients with acute pericarditis, as well as the significance of factors involved in pericardial T1 mapping measurements. To better capture the inflammatory process during the acute phase of pericarditis, which consistently affects the pericardial layers but is frequently, though not always, accompanied by pericardial effusion, we proposed a new method for T1 mapping region of interest (ROI) measurement. This method is not limited to the pericardial effusion but includes all pericardial structures within the pericardial layers, encompassing both the pericardial effusion and the thickness of the pericardial layers.

Therefore, the first aim of the study was to investigate the impact of the native T1 values of the pericardium as a possible parameter for a contrast-free diagnosis of acute pericarditis. We also aimed to explore the association of pericardial T1 mapping with demographic, cardiovascular risk factors, clinical parameters, cardiac biomarkers, and cardiac function.

## 2. Materials and Methods

### 2.1. Study Population

In this retrospective, cross-sectional, observational, single-center study, all patients presenting with acute pericarditis who underwent CMR between March 2017 and March 2024 were included. Eligible patients met the following criteria: (1) a clinical diagnosis of the first episode of acute pericarditis according to the Position Statement of the European Society of Cardiology Heart Failure Association [2], defined by at least two of four criteria: pericardial chest pain, a pericardial friction rub, newly observed diffuse ST-segment elevation or PR-segment depression on an electrocardiogram, and the emergence or worsening of a pericardial effusion, and (2) the availability of a CMR examination within 7 days after symptom onset.

Exclusion criteria included patients under 18 years of age; previous myocardial infarction; signs of myocardial involvement on CMR according to the updated Lake Louise Criteria [12], pre-existing cardiomyopathy; a prior history of atrial fibrillation; chronic and/or recurrent pericarditis; sub-optimal or incomplete CMR images; and suspected or known prior irreversible myocardial damage.

Controls were age- and sex-matched, and underwent CMR to rule out scar-related ventricular tachycardia. Exclusion criteria for the control group included patients under 18 years of age; signs of a structural heart defect on CMR; known liver, renal, or systemic inflammatory diseases; and cases where a complete CMR protocol, including T1 mapping, was not performed.

Cardiovascular risk factors, clinical data, and bio-humoral markers were collected from hospital records during the initial hospitalization. Hypertension was defined as a systolic blood pressure of ≥140 mmHg or a diastolic blood pressure of ≥90 mmHg at rest on more than two occasions, or the use of antihypertensive drugs [13]. Smoking status was defined as current smokers or never smokers. Cholesterol laboratory analyses were conducted following the standard in-house protocol. Diabetes status was assessed using the World Health Organization criteria [14] or an established diagnosis of type 2 diabetes. Obesity was defined as a BMI > 30, as defined by the World Health Organization criteria [15]. Bio-humoral markers collected in this retrospective study included white blood cell count, C-reactive protein (CRP), hs-troponin I, and erythrocyte sedimentation rate (ESR).

The study received approval from the Institutional Review Board, and patient consent was waived due to the retrospective nature of the study.

**Figure 1:**
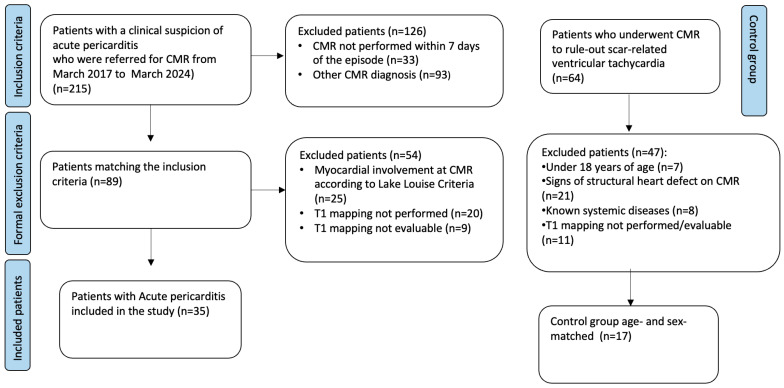
Flowchart of the patients enrolled.

A flowchart depicting the inclusion and exclusion criteria application is provided in Figure 1.

### 2.2. CMR Acquisition

CMR scans were performed at 3.6 ± 2.8 days (median = 1 day, range = 1–7 days) after hospital admission using a Philips Achieva dStream 1.5-T scanner system (Philips Medical Systems, Best, The Netherlands) with anterior 32-channel phased array coils. Cine images were acquired using balanced steady-state free precession and retrospective gating during expiratory breath-hold maneuvers (TE = 1.7 ms; TR = 3.4 ms; flip angle = 45°; section thickness = 8 mm) in both long-axis (two-, three-, and four-chamber views) and short-axis planes. Short-axis stacks covered the whole LV with the same center of slice as 5-into-3 planning.

T1 mapping was performed in the short-axis plane in three slices (at the base, mid-ventricular, and apex) using a single-breath-hold, ECG-triggered modified look-locker inversion recovery 5s(3s)3s acquisition scheme before contrast media injection (TE = 1.12 ms; TR = 2.5 ms; flip angle = 35°; FOV = 300 × 300 mm).

LGE imaging was conducted in both long- and short-axis slices 10–12 min after contrast media injection (Gadovist, Bayer Healthcare, Leverkusen, Germany) at a dose of 0.15 mL per kg body weight using phase-sensitive inversion recovery sequences (TE = 2.0 ms; TR = 3.4 ms; flip angle = 20°; section thickness = 8 mm) with the inversion time determined by the Look-Locker technique.

### 2.3. CMR Image Post-Processing

**Figure 2:**
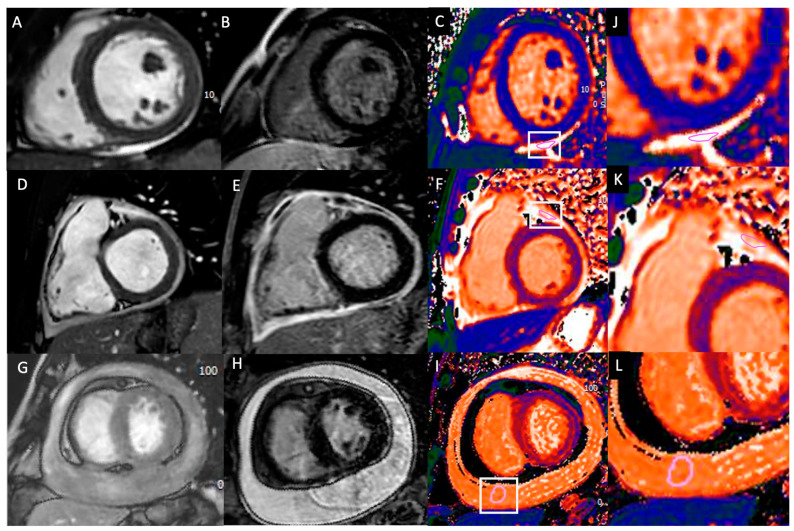
Figure legends: Case examples of pericardial T1 mapping are presented for a control subject (**A**–**C**), a patient with acute pericarditis without significant pericardial effusion (**D**–**F**), and patients with acute pericarditis and moderate pericardial effusion (**G**–**I**). Zoomed-in T1 mapping images are also provided (**J**–**L**).

Pericardial T1 mapping analysis was performed using dedicated CMR software (CV42 6.0, CVI42, Circle Cardiovascular Imaging Inc., Calgary, AB, Canada) through a ROI within the pericardium. An experienced observer (R.C., with seven years of experience in cardiovascular imaging), blinded to the patients’ conditions, manually delineated a ROI with a size > 10 mm^2^ and <50 mm^2^ in the pericardium. The pericardial T1 measurements were obtained by tracing a freehand ROI according to the morphology of the pericardium at the point of maximum thickness. To ensure the quality of the measurements and to avoid an excessively small ROI, as suggested by prior study [16], the ROI included all pericardial structures confined within the pericardial layers, encompassing both pericardial effusion and pericardial layers thickness, while avoiding the inclusion of extrapericardial structures (Figure 2).

Figure 2A shows a short-axis SSFP image of a control subject with no significant pericardial effusion. Figure 2D illustrates circumferential pericardial thickening in a patient with acute pericarditis but no significant effusion. Figure 2G depicts moderate circumferential pericardial effusion in a patient with acute pericarditis. Short-axis LGE images highlight significant pericardial enhancement (LGE) in patients with acute pericarditis, with signal intensity exceeding that of the ventricular blood pool (Figure 2E,H). In contrast, no pericardial enhancement is seen in the control subject (Figure 2B). Freehand regions of interest (ROIs) measuring 10–50 mm² were placed along the pericardial structure on the T1 mapping images (purple circles in Figure 2C,F,I). Zoomed-in T1 mapping images with ROI measurements are shown in Figure 2J–L.

Myocardial T1 mapping values were generated offline using the same dedicated CMR software. Epi- and endocardial borders were manually traced, propagated through the image stack, and corrected manually when necessary.

Pericardial effusion and thickness quantification were quantified by directly delineating the pericardium on cine-CMR images in the end-diastolic short-axis view, measuring its maximal extent [17].

The presence of LGE in the pericardium was evaluated using both qualitative and semiquantitative methods [18]. Briefly, LGE in the pericardium was semiquantitative categorized as follows: none (no apparent LGE visible), mild (subtle LGE in the pericardium with signal intensity lower than that of the ventricular blood pool), moderate (clear enhancement similar to the ventricular blood pool), or severe (significant and visually prominent LGE in the pericardium with signal intensity higher than that of the ventricular blood pool) [18].

### 2.4. Reproducibility

To assess intra-observer variability, an experienced observer re-evaluated a random subset of 20 patients, including both cases of acute pericarditis and control subjects, within a minimum interval of one week. For interobserver analysis, a second blinded observer (M.P., with six years of experience in cardiovascular imaging), who was unaware of the initial results, performed the same post-processing analysis on the same random subset of 20 patients.

### 2.5. Statistical Analysis

Continuous variables were presented as mean (standard deviation [SD]), while categorical variables were expressed as frequency (%). Comparisons of continuous variables were conducted using Welch’s *t*-test, with Kolmogorov–Smirnov tests employed to assess the normality of residuals.

The Kruskal–Wallis *H* test with Bonferroni test was used for comparisons of multiple groups. Categorical variables were analyzed using the chi-square test or Fisher’s exact test, as appropriate.

Correlation was assessed using the Pearson r and Spearman rho coefficient as appropriate. Association between T1 mapping in the pericardium, clinical parameters, and CMR features were analyzed using multivariate linear regression.

The receiver operating characteristic (ROC) analysis was applied to estimate the diagnostic value of T1 mapping parameters for detecting acute pericarditis. The results were presented as areas under the curve (AUCs) with 95% confidence intervals (CIs) and the optimal cut-off value was calculated using the Youden index method.

All statistical tests were two-sided, and a *p*-value < 0.05 was considered statistically significant. All statistical analyses were performed using JASP Statistics 0.18.3.

## 3. Results

### 3.1. Baseline Characteristics

A total of 35 patients with acute pericarditis, comprising 26 males (74%) and 9 females (26%) with a mean age of 45.54 ± 23.38 years, were included. Seventeen control subjects, comprising 12 males (71%) and 5 females (29%) with a mean age of 47.78 ±19.38 years, were also included.

No significant differences were found in age and sex among the enrolled patients (*p* = 0.722 and *p* = 0.783, respectively). Furthermore, no significant differences in cardiovascular risk factors were observed between the enrolled groups. In acute pericarditis patients, leukocytosis was observed in 13 (37%) patients, whereas high C-reactive protein and high erythrocyte sedimentation rates were present in 29 (83%) and 12 (34%), respectively.

Table 1 shows the demographic and clinical characteristics of acute pericarditis patients.

Among the acute pericarditis patients enrolled, 29 had idiopathic pericarditis and 6 had non-infectious causes of pericarditis, including 4 with connective tissue diseases and 2 with a history of previous radiotherapy.

### 3.2. CMR Features in Acute Pericarditis Patients

**Figure 3:**
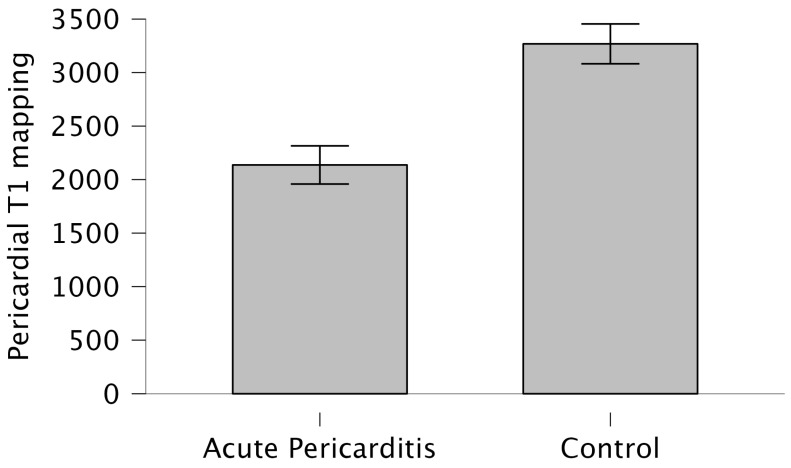
Pericardial T1 mapping in acute pericarditis and control controls.

CMR characteristics of patients enrolled are summarized in Table 2. No significant differences were found in left ventricle and right ventricle volumes and functions. Patients with acute pericarditis showed a median pericardial thickness of 4.74 ± 8.71 mm. Among the acute pericarditis patients enrolled, 23 (66%) demonstrated the presence of pericardial LGE enhancement. Specifically, six (17%) patients showed mild pericardial enhancement, ten (28%) moderate pericardial enhancement, and seven (20%) severe pericardial enhancement. Compared to control subjects, acute pericarditis patients exhibited significantly lower pericardial T1 mapping values (2137 ± 519 vs. 3268 ± 362, *p* = 0.001) (Figure 3).

Pericardial T1 mapping values decrease as pericardial enhancement increases (*p* = 0.006). Acute pericarditis patients without pericardial LGE enhancement show T1 pericardial values of 2574 ± 411 ms. In contrast, patients with mild LGE enhancement have values of 2498 ± 20 ms, those with moderate LGE enhancement have values of 2066 ± 451 ms, and those with severe pericardial enhancement have values of 1628 ± 273 ms (Supplemental Figure S1).

Conversely, no significant differences in myocardium T1 mapping were observed between acute pericarditis patients and control subjects (1032 ± 155 vs. 1027 ± 63, *p* = 0.913).

### 3.3. Demographic, Clinical, Bio-Humoral Markers, and CMR Features Correlates of Pericardial T1 Mapping Values in Acute Pericarditis Patients

**Figure 4:**
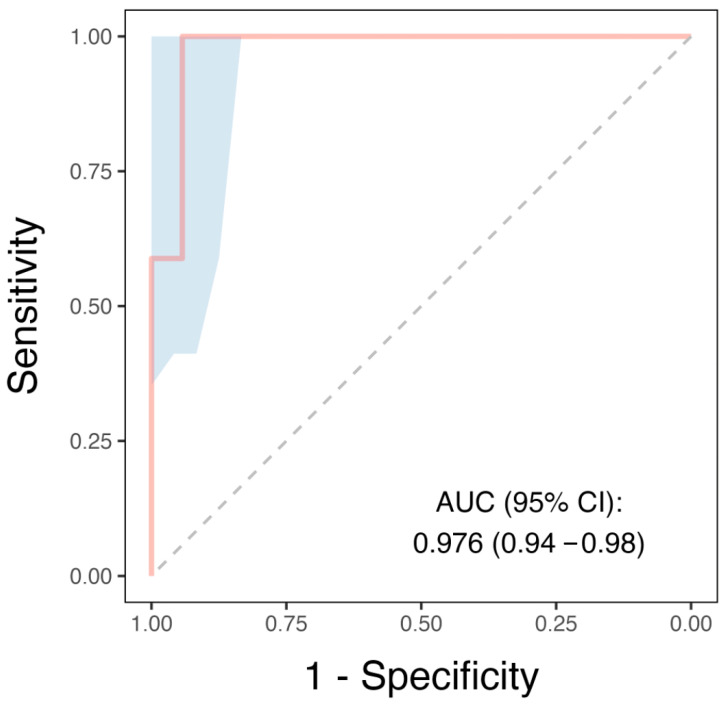
ROC curve analysis of T1 mapping to detect acute pericarditis. The ROC curve shows the diagnostic performance of pericardial T1 mapping in distinguishing between patients with acute pericarditis and control patients. The curve plots sensitivity (true positive rate) against 1 − specificity (false positive rate) at various T1 mapping values. The blue shading represents the 95% confidence interval, computed using a bootstrap method, which indicates the uncertainty associated with the estimated sensitivity and specificity values.

Univariable and multivariable analyses are presented in Table 3. Univariable analysis revealed that hypertension, erythrocyte sedimentation rate, and grading of pericardial enhancement were independently associated with pericardial T1 mapping values (β coefficient = −2.107, *p* = 0.040; β coefficient = −2.204, *p* = 0.035; and β coefficient = −3.625, *p* = 0.001, respectively). Further multivariable analysis revealed that the grading of pericardial enhancement was the only independent determinant of pericardial T1 mapping values (β coefficient = −3.271, *p* = 0.003). Analysis of the ROC curve (Figure 4) showed an area under the curve of 0.97 (95% CI = 0.94–0.98), with a best pericardial T1 mapping cut-off of 2862.5 ms for the detection of acute pericarditis (*p* = 0.001). This cut-off had a sensitivity of 99% and a specificity of 94%.

### 3.4. Reproducibility

The intraobserver and interobserver agreement for T1 mapping parameters was good. The intraclass correlation coefficients ranged from 0.893 to 0.964 for intraobserver agreement, and from 0.888 to 0.920 for interobserver analysis.

## 4. Discussion

In the present study, we demonstrated a significant difference in T1 mapping values between patients with acute pericarditis and control subjects. Notably, among the acute pericarditis group, the intensity of LGE in the pericardium emerged as the only independent determinant of pericardial T1 mapping values. Furthermore, our findings suggest that pericardial T1 mapping by CMR could serve as a valuable tool for distinguishing acute pericarditis from control subjects, achieving an AUC of 0.97 for the diagnosis of acute pericarditis.

CMR represents a crucial imaging modality in the diagnostic evaluation of various cardiovascular diseases, providing a non-invasive assessment of cardiac structure, volume, function, and myocardial tissue characteristics [19,20,21]. Using specific imaging biomarkers, CMR has been shown in numerous studies to discriminate among different cardiovascular disease etiologies [22,23,24,25]. The advanced tissue characterization offered by CMR, including techniques such as T1 mapping, has broadened its utility beyond anatomical imaging, providing insights into pathophysiological mechanisms and enhancing CMR’s role in personalized patient care [6,26,27,28,29].

Previous studies have investigated both in vivo and in vitro the use of T1 mapping to differentiate the composition of pericardial effusion, demonstrating distinct T1 mapping values in exudative and transudative effusions. [11,30,31,32].

In the clinical setting of acute pericarditis [33], an inflammatory condition that causes the pericardial sac to produce exudate containing fluid, fibrin, and cells [33], there are resulting changes in pericardial effusion composition and variations in pericardial T1 mapping values. This occurs because the paramagnetic properties of proteins and cells within the exudate lead to a reduction in T1 mapping values [30,34]. Conversely, in control subjects, higher pericardial T1 values reflect the presence of transudative effusion, consistent with findings from previous studies [11,30].

It is also important to highlight that T1 mapping measures the longitudinal, or spin-lattice, relaxation time, which varies depending on the tissue composition, thereby influencing T1 mapping values [6]. When measuring T1 mapping in the pericardium, key biological determinants to consider are water, fat, and fibrous tissue. Increased water content, such as in pericardial effusion, has a long T1 relaxation time and generally leads to elevated T1 values [6]. The T1 values of fibrous tissue, on the other hand, vary depending on the presence of inflammation and its composition, typically showing lower T1 values compared to water. In acute pericarditis, thickened and inflamed pericardial layers result in lower T1 values compared to pericardial effusion [6,11,26]. It is also important to consider extrapericardial structures, such as pericardial fat, which should be included in the ROI measurement, particularly in cases with minimal pericardial effusion. Fat has very low T1 values (230–350 ms at 1.5 T), and its inclusion can significantly influence the overall T1 mapping results [6].

Unlike previous studies, our research is unique in being the first to evaluate the diagnostic performance of non-contrast T1 mapping as part of a comprehensive CMR imaging protocol specifically in patients presenting with clinical suspicion of acute pericarditis [6,16,35,36].

### 4.1. Clinical Implications

In clinical practice, transthoracic echocardiography is the first-line imaging modality for the evaluation of pericardial conditions due to its availability, portability, and low cost. However, despite the widespread accessibility of echocardiography, CMR has emerged as an important imaging tool for the diagnosis and management of pericarditis [37]. Although CMR has limited availability and is associated with higher costs compared to echocardiography, recent international position statements have emphasized its growing role in improving diagnostic accuracy [37]. Thanks to its tissue characterization capabilities, CMR can serve as a non-invasive method for characterizing pericardial fluid, potentially avoiding the need for diagnostic pericardiocentesis in cases of transudate.

In this scenario, the rapid increase in CMR examinations highlights the need to optimize clinical workflows with faster and more cost-effective protocols. Additionally, shorter, non-contrast CMR examinations are beneficial for individuals ineligible for contrast media and those with limited tolerance for lengthy procedures. Non-contrast CMR imaging enhances the applicability of CMR, improves patient comfort, and reduces costs. Our results demonstrate that pericardial T1 mapping may serve as a helpful and supportive non-contrast CMR parameter in the diagnostic work of patients with acute pericarditis.

### 4.2. Future Perspective

Despite promising results regarding the role of T1 mapping in the clinical evaluation of acute pericarditis, variations in protocols, sequences, scanner types, and field strengths across institutions have led to inconsistencies in measurements. Establishing standardized acquisition and analysis protocols is essential for reliable comparisons across studies and clinical settings. Further multi-center studies are needed to establish robust reference values across diverse populations and scanner types to support its routine clinical use. Additionally, multi-parametric CMR approaches, combining T1 mapping with T2 mapping and extracellular volume (ECV) quantification, may enhance diagnostic precision by providing a comprehensive assessment of tissue characteristics in patients with acute pericarditis, as well as identifying concomitant myocardial involvement. While T1 mapping has demonstrated significant sensitivity as a marker for various acute myocardial diseases, refs. [6,16] its ability to distinguish between acute, sub-acute, and chronic inflammatory processes remains limited [35]. This limitation arises because T1 mapping values can be affected by overlapping features common to both acute and chronic stages, such as edema and fibrosis, which are present across different phases of myocardial inflammation [6]. In the context of acute pericarditis, although T1 mapping is effective at detecting inflammation, it may not reliably differentiate between acute, sub-acute, and chronic stages. This highlights the need for an integrated assessment using additional protocols, including T2 mapping and ECV measurement, to achieve more precise differentiation among these stages of pericarditis [36].

### 4.3. Limitations

This study has several limitations that should be acknowledged. First, the sample size is relatively small, and the study is retrospective in nature, which may limit the generalizability of the findings and reduce the statistical power of certain analyses, particularly the ROC curve assessment. Due to the limited number of participants and the absence of specific types of pericarditis, our results may not fully represent the broader population of patients with acute pericarditis. Future research with larger cohorts is necessary to confirm our findings, address the potential biases associated with a small sample size, and evaluate differences in T1 mapping values across various etiologies of acute pericarditis. Another limitation of this study is the use of a control group consisting of patients who underwent CMR to rule out scar-related ventricular tachycardia. The heart rate dependence of the T1 mapping MOLLI sequence may introduce variability, as higher heart rates can lead to incomplete relaxation and residual longitudinal magnetization at the time of the next inversion, resulting in lower measured T1 values.

Furthermore, we tracked the ROI in the pericardium only in the short-axis view, as our T1 mapping study protocol did not include other views. The size of the ROI, particularly when assessing normal pericardial fluid in control subjects, may result in partial volume effects and the potential inclusion of extrapericardial structures, such as fat. A more comprehensive assessment of T1 mapping values for pericardial structures could be achieved by utilizing dedicated and additional slice orientations. Finally, this study did not evaluate longitudinal changes in T1 mapping values or their predictive value for long-term outcomes in patients with acute pericarditis, which could be an important area for future research

## 5. Conclusions

Pericardial T1 mapping emerges as a promising non-contrast CMR parameter for diagnosing acute pericarditis, demonstrating high diagnostic accuracy and a strong association with the severity of pericardial late gadolinium enhancement. The findings suggest that T1 mapping could be integrated into clinical practice as a valuable tool for the non-invasive evaluation of pericardial inflammation, particularly in patients who are unable to receive contrast agents. However, further studies with larger, more diverse populations and comprehensive imaging protocols are needed to validate these results and explore the full potential of T1 mapping in differentiating various stages and types of pericardial and myocardial inflammation. If confirmed in future research, these findings could lead to more efficient, patient-friendly diagnostic pathways in the management of pericarditis.

## Figures and Tables

**Table 1:** Baseline characteristics of patients and normal controls.

Variables	Acute Pericarditis	Control	*p*-Values
**Sex (male), n (%)**	26 (74%)	12 (71%)	0.783
**Age (years)**	45.54 ± 23.38	47.78 ± 19.38	0.722
**Hypertension, n (%)**	15 (43%)	3 (18%)	0.064
**Dyslipidemia, n (%)**	5 (14%)	3 (17%)	0.791
**Smoke, n (%)**	8 (23%)	4 (23%)	0.981
**Obesity, n (%)**	5 (14%)	1 (6%)	0.367
**Diabetes, n (%)**	3 (9%)	2 (12%)	0.745
**Familiary for CAD, n (%)**	9 (22%)	3 (18%)	0.815
**Pericarditis chest pain, n (%)**	32 (91%)	/	/
**Pericardial rubs, n (%)**	17 (48%)	/	/
**ECG abnormalities, n (%)**	21 (60%)	/	/
**Pericardial effusion, n (%)**	26 (74%)	/	/
**Leukocitosis, n (%)**	13 (37%)	/	/
**CRP, n (%)**	29 (83%)	/	/
**Erythrocyte sedimentation rate, n (%)**	12 (34%)	/	/
**Fever, n (%)**	15 (43%)	/	/
**Troponin, n (%)**	7 (20%)	/	/

Abbreviations: CAD coronary artery disease; CRP, C-reactive protein.

**Table 2:** CMR characteristics of patients and normal controls.

Variables	Acute Pericarditis	Control	*p*-Values
**LVEF, %**	57.18 ± 5.8	60.26 ± 5.76	0.157
**LVEDV/BSA, mL/m^2^**	86.18 ± 23	82.23 ± 18.72	0.551
**LVESV/BSA, mL/m^2^**	36.39 ± 14.70	33.63 ± 10.34	0.506
**LVSV/BSA, mL/m^2^**	49.82 ± 10.81	48.58 ± 9.24	0.695
**RVEF, %**	56.38 ± 6.47	56.72 ± 5.5	0.857
**RVEDV/BSA, mL/m^2^**	78.70 ± 16.18	78.58 ± 11.45	0.982
**RVESV/BSA, mL/m^2^**	35.04 ± 11.17	35.09 ± 11.97	0.987
**RVSV/BSA, mL/m^2^**	43.82 ± 9.04	43.48 ± 10.19	0.905
**Pericardial thickness, mm**	4.74 ± 8.71 [0–34]	/	/
**Pericardial effusion thickness, mm**	7.87 ± 13.53 [0–32]	2.88 ± 2.05 [1–5]	**0.001**
**LGE pericardial enhancement, n (%)**	23 (66%)	/	/
**LGE grading, n (%)**			
**No pericardial LGE**	11 (31%)	/	/
**Mild pericardial LGE**	6 (17%)	/	/
**Moderate pericardial LGE**	10 (28%)	/	/
**Severe pericardial LGE**	7 (23%)	/	/
**Pericardial T2 STIR, n (%)**	28 (80%)	/	/
**Myocardium T1 mapping, ms**	1032 ± 155	1027 ± 63	0.913
**Pericardial T1 mapping, ms**	2137 ± 519	3268 ± 362	**0.001**

Bold indicates statistical significance. Abbreviations: BSA, body surface area; EDV, end-diastolic volume; ESV, end-systolic volume; LGE, late gadolinium enhancement; LV, left ventricle; STIR, short tau inversion recovery; SV, stroke volume; RV, right ventricle.

**Table 3:** Univariable and multivariable determinants of pericardial T1 mapping in acute pericarditis patients.

Variables	Univariable		Multivariable	
	*β Coefficent*	*p Values*	*β Coefficent*	*p Values*
**Sex**	1.066	0.292		
**Age**	−0.698	0.488		
**Hypertension**	−2.107	**0.040**	−0.989	0.331
**Dyslipidemia**	0.646	0.521		
**Smoke**	−0.898	0.373		
**Obesity**	−1.458	0.151		
**Diabetes**	−0.800	0.428		
**Familiary for CAD**	−1.776	0.082		
**Pericardial thickness**	−0.258	0.798		
**Leukocitosis**	−0.856	0.398		
**CRP**	−0.427	0.672		
**Erythrocyte sedimentation rate**	−2.204	**0.035**	−0.987	0.336
**Fever**	−0.361	0.720		
**Troponin**	0.459	0.649		
**LVEF**	0.794	0.431		
**LVEDV/BSA**	0.861	0.394		
**LVESV/BSA**	−0,299	0.766		
**LVSV/BSA**	−0.934	0.355		
**RVEF**	−0.650	0.519		
**RVEDV/BSA**	0.859	0.395		
**RVESV/BSA**	−1.048	0.300		
**RVSV/BSA**	−0.517	0.608		
**T2 STIR**	−1.700	0.099		
**LGE presence**	−1.686	0.101		
**LGE grading**	−3.625	**0.001**	−3.271	**0.003**
**T1 mapping (myocardium)**	−0.144	0.886		

Multivariable analysis was adjusted for factors that were statistically significant in the univariable analysis. Bold indicates statistical significance. BSA, body surface area; CAD coronary artery disease; EDV, end-diastolic volume; ESV, end-systolic volume; LGE, late gadolinium enhancement; LV, left ventricle; STIR, short tau inversion recovery; SV, stroke volume; RV, right ventricle.

## Data Availability

Data will be made available upon reasonable request to the corresponding authors.

## Supplementary Materials

The following supporting information can be downloaded at: https://www.mdpi.com/article/10.3390/tomography10120137/s1, Figure S1: Pericardial T1 mapping values stratified by pericardial enhancement.
