# Supplementary figures and images for: Assessing Acute Pericarditis with T1 Mapping: A Supportive Contrast-Free CMR Marker
Source: Tomography. 2024 Nov 27;10(12):1881–94. doi: 10.3390/tomography10120137 (PMC11679063; doi:10.3390/tomography10120137)

Figure S1: Pericardial T1 mapping values stratified by pericardial enhancement.

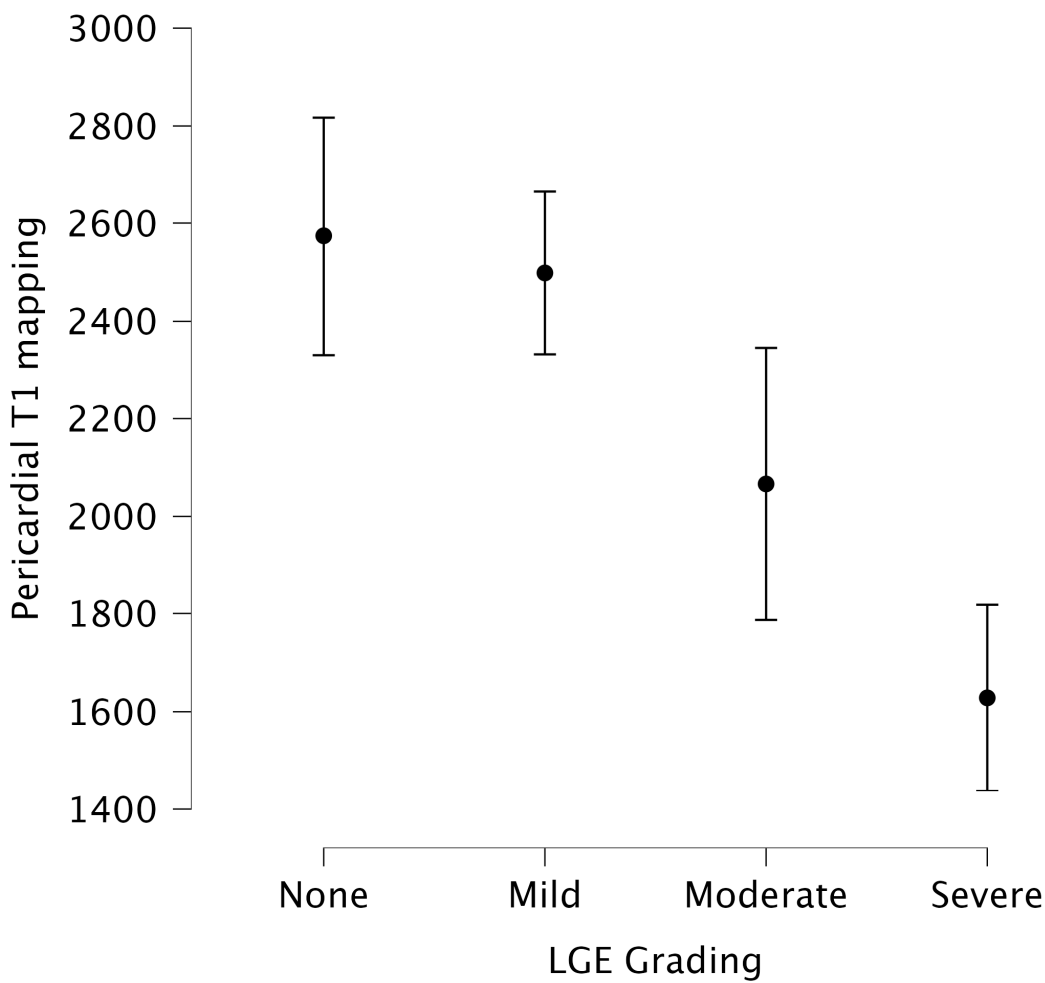

Supplement: Supplementary file 1 [file tomography-10-00137-s001.zip › tomography-3304293-supplementary.pdf]
